# Bone Mineral Density After Transitioning From Denosumab to Alendronate

**DOI:** 10.1210/clinem/dgz095

**Published:** 2019-10-26

**Authors:** David Kendler, Arkadi Chines, Patricia Clark, Peter R Ebeling, Michael McClung, Yumie Rhee, Shuang Huang, Robert Kees Stad

**Affiliations:** 1 University of British Columbia, Vancouver, BC, Canada; 2 Amgen Inc., Thousand Oaks, CA, US; 3 Hospital Infantil de Mexico Federico Gómez and National University of Mexico-UNAM, Mexico City, Mexico; 4 Monash University, Melbourne, VIC, Australia; 5 Oregon Osteoporosis Center, Portland, OR, US; 6 Australian Catholic University, Melbourne, VIC, Australia; 7 Yonsei University College of Medicine, Seoul, South Korea; 8 Amgen Inc., Rotkreuz, Switzerland

**Keywords:** denosumab, alendronate, bisphosphonates, bone mineral density, osteoporosis

## Abstract

**Context:**

There are few studies on patients transitioning from denosumab to bisphosphonates.

**Objective:**

To investigate patient characteristics and changes in bone mineral density (BMD) after transitioning from denosumab to alendronate.

**Design:**

Randomized, open-label, 2-year crossover Denosumab Adherence Preference Satisfaction (DAPS) study (NCT00518531).

**Setting:**

25 study centers in the US and Canada.

**Patients:**

Treatment-naïve postmenopausal women with BMD T-scores from −2.0 to −4.0.

**Interventions:**

This post hoc analysis evaluated women randomized to subcutaneous denosumab 60 mg every 6 months in year 1 followed by once-weekly oral alendronate 70 mg in year 2.

**Main Outcome Measure:**

A 3% BMD threshold identified participants who lost, maintained, or gained BMD in year 2 on alendronate.

**Results:**

Of 126 participants randomized to denosumab, 115 (91%) transitioned to alendronate in year 2. BMD increased by 3% to 6% with denosumab in year 1 and by 0% to 1% with alendronate in year 2. After transitioning to alendronate, most participants maintained or increased BMD; 15.9%, 7.6%, and 21.7% lost BMD at the lumbar spine, total hip, and femoral neck, respectively. Few participants fell below their pretreatment baseline BMD value; this occurred most often in those who lost BMD in year 2. Women who lost BMD with alendronate in year 2 also showed a greater percent change in BMD with denosumab in year 1. The BMD change in year 2 was similar regardless of baseline characteristics or adherence to oral alendronate.

**Conclusion:**

Alendronate can effectively maintain the BMD gains accrued after 1 year of denosumab in most patients, regardless of baseline characteristics.

Postmenopausal osteoporosis is a chronic disease associated with age-related declines in bone mass, changes in bone microarchitecture, and skeletal fragility. These changes place postmenopausal women at increased risk of fragility fractures, which are linked to significant morbidity, economic cost, and negative impact on health-related quality of life (1–4). Antiresorptive therapies, including bisphosphonates and denosumab, have been demonstrated to reduce fracture incidence and increase bone mineral density (BMD) in postmenopausal women with osteoporosis (5–9), although the optimal duration and sequencing of available treatments remain poorly understood. Whereas bisphosphonates bind to bone mineral and become incorporated into bone matrix, denosumab, a monoclonal antibody targeting receptor activator of nuclear factor kappa-B ligand (RANKL), is a reversible therapy. There is a rebound in bone turnover after treatment cessation, leading to loss of the bone density gained on treatment and loss of vertebral fracture protection (10–12). If denosumab is discontinued, follow-on therapy with a bisphosphonate has been recommended to prevent reversible bone loss (13, 14), although limited data are available on patients transitioning from denosumab to bisphosphonates, such as alendronate.

The Denosumab Adherence Preference Satisfaction (DAPS) study was a 24-month study designed to compare adherence to denosumab with alendronate over 12 months in postmenopausal women with low BMD. Participants were randomized to receive denosumab or alendronate for the first year, after which they crossed over to receive the other treatment for the second year. We previously reported that the primary efficacy endpoints of adherence, preference, and satisfaction favored injectable denosumab over oral alendronate and that alendronate could maintain the gains in BMD achieved with 1 year of denosumab treatment (15, 16). Here we perform a descriptive subanalysis of participants randomized to the denosumab/alendronate sequence to investigate the effect of transitioning to alendronate.

## Materials and Methods

### Study design

The DAPS study (NCT00518531) was a 24-month, multicenter, randomized, open-label, crossover study conducted at 20 centers in the US and 5 centers in Canada, from October 2007 to July 2010. Study details have been previously described (15, 16). Briefly, participants were randomized 1:1 to receive 1 of 2 treatment sequences: denosumab followed by alendronate or alendronate followed by denosumab. The current analysis focused on the denosumab/alendronate sequence, in which subjects received subcutaneous denosumab, 60 mg every 6 months, in the first year and then crossed over to oral alendronate, 70 mg once weekly, in the second year. All participants received daily supplementation of calcium (1000 mg) and vitamin D (at least 400 IU). Women who withdrew from treatment during the first year but wished to remain in the study were allowed to crossover early to the second year of treatment.

### Eligibility criteria

Enrolled participants were ambulatory postmenopausal women aged 55 years or older with baseline BMD T-scores from −4.0 to −2.0 at the lumbar spine (LS), total hip (TH), or femoral neck (FN), as measured using dual-energy x-ray absorptiometry (DXA). Patients were excluded if they had received prior bisphosphonate or denosumab treatment or bone-acting drugs, including glucocorticoids. Additional exclusion criteria included hyper/hypocalcemia, vitamin D deficiency (< 20 ng/mL [49.9 nmol/L]), or contraindications to alendronate treatment. Written informed consent was obtained from each participant, and this study was conducted in accordance with the principles set out in the Declaration of Helsinki and was formally approved by the appropriate institutional review board, ethical review committee, or equivalent at each study site.

### Outcome measures

BMD at the LS, TH, and FN was measured by DXA at baseline (day 1 visit) and at months 12 and 24 of the treatment period. DXA scans were performed at the local study sites, and the same DXA machine was used for all study procedures for a particular participant. Bone turnover markers (BTMs), including fasting serum C-telopeptide (CTX-1) and N-terminal propeptide type I procollagen (P1NP), were assessed at baseline and months 12, 18, and 24 of treatment. Adherence to oral alendronate was defined as a composite endpoint of A) taking ≥ 80% of weekly alendronate tablets (overall treatment compliance) and at least 2 tablets in the last month (treatment persistence), as monitored using Medication Event Monitoring System (MEMS) technology, and B) completing the relevant treatment period. We evaluated participant characteristics including baseline age, history of fracture, baseline BMD, change in BMD at months 12 and 24, change in BTM at months 12 and 24, and adherence to alendronate.

### Statistical analyses

BMD and BTM values were summarized using descriptive statistics and plotted as the mean with 95% confidence interval (CI) and median with interquartile range (IQR), respectively. BMD change during the study period was also plotted at the level of individual participants. Descriptive analysis was performed to evaluate baseline and month 12 and 24 characteristics in groups of participants that lost, maintained, or gained BMD after transitioning from denosumab to alendronate in year 2, defined using a 3% BMD least significant change threshold. The 3% threshold was selected according to DXA scanner precision of approximately 1% corresponding to an approximate least significant change in BMD of 3%, as applied previously in responder analyses of drugs used to treat osteoporosis or increase BMD (17–21). For LS BMD assessment, participants were excluded if there were fewer than 4 evaluable vertebrae. A change in BMD ≤ −3% indicated lost BMD, change from > −3% to < 3% indicated maintained BMD, and change ≥ 3% indicated gained BMD. This grouping was applied separately for each skeletal site. All analyses were descriptive in nature. A summary of incidence of adverse events was generated for each treatment year.

## Results

### Participant characteristics

The DAPS study enrolled 250 participants. Of 126 women randomized to the denosumab/alendronate sequence, 114 (90.5%) completed denosumab treatment in year 1 and 115 (91%) transitioned to alendronate at month 12, including 3 who crossed over early. Alendronate treatment in year 2 was completed by 95 participants (82.6%). Most common reasons for discontinuation before completion of treatment included withdrawn consent (6 participants [4.8%] in year 1; 8 [7.0%] in year 2), lost to follow-up (3 [2.4%] in year 1; 0 [0.0%] in year 2), and adverse events (0 [0.0%] in year 1; 7 [6.1%] in year 2) (Table 1). Characteristics of the 126 participants initiating denosumab treatment in year 1 and the 115 participants initiating alendronate treatment in year 2 are shown in Table 2. At baseline, participants had a mean age of 65 years and mean BMD T-scores of −2.0, −1.6, and −2.0 at the LS, TH, and FN, respectively. Prior osteoporotic fracture was reported for 37.3% of participants at baseline. Similar characteristics were observed for the participants who transitioned to alendronate at month 12.

**Table 1.** Numbers of Participants Completing Each Treatment Period and Reasons for Study Discontinuation

	Denosumab/Alendronate Sequence	
	Denosumab Year 1 (N = 126)	Alendronate Year 2 (N = 115)
Completed treatment period, n (%)	114 (90.5)	95 (82.6)
Discontinued before completing treatment period, n (%)	12 (9.5)	20 (17.4)
Early crossover	3 (2.4)	N/A
Adverse event	2 (1.6)	N/A
Administrative decision	1 (0.8)	N/A
Early termination	9 (7.1)	20 (17.4)
Consent withdrawn	6 (4.8)	8 (7.0)
Lost to follow-up	3 (2.4)	0 (0.0)
Adverse event	0 (0.0)	7 (6.1)
Complete out of scheduled visit window	0 (0.0)	2 (1.7)
Noncompliance	0 (0.0)	2 (1.7)
Protocol-specified criteria	0 (0.0)	1 (0.9)

N = number of participants randomized (year 1) or crossed over (year 2); n = number of participants with the characteristic of interest; N/A = not applicable.

**Table 2.** Participant Characteristics at Study Baseline

	Denosumab/Alendronate Sequence	
	Denosumab Year 1 (N = 126)	Alendronate Year 2 (N = 115)
Race/ethnicity—white, n (%)	115 (91)	107 (93)
Age, mean (SD), years	65.1 (7.6)	65.1 (7.4)
Years since menopause, mean (SD)	18.2 (11.4)	17.9 (10.9)
BMD T-score at the start of each year, mean (SD)		
Lumbar spine	–2.04 (1.16)	–1.61 (1.29)
Total hip	–1.60 (0.74)	–1.38 (0.74)
Femoral neck	–2.01 (0.55)	–1.84 (0.6)
Prior osteoporotic fracture, n (%)	47 (37.3)	41 (35.7)

N = number of participants randomized (year 1) or crossed over (year 2); n = number of participants with the characteristic of interest.

Abbreviations: BMD, bone mineral density SD, standard deviation.

### Change in BMD and BTM with denosumab in year 1 and alendronate in year 2

**Figure 1.**
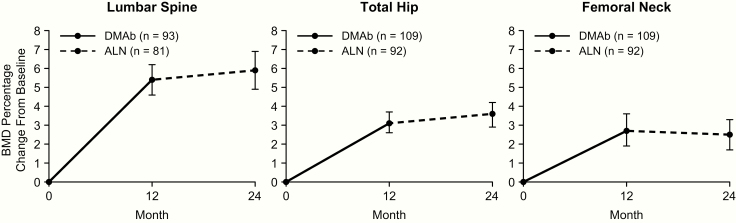
BMD percentage change from baseline with denosumab in year 1 and alendronate in year 2. Data show mean and 95% confidence interval. n = number of participants with measurements at baseline and month 12 (DMAb) or month 24 (ALN). Abbreviations: ALN, alendronate; BMD, bone mineral density; DMAb, denosumab.

**Figure 2.**
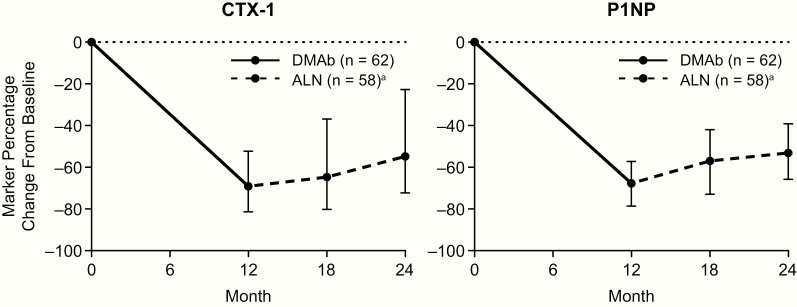
BTM percentage change from baseline with denosumab in year 1 and alendronate in year 2. Data show mean and interquartile range. n = number of participants with measurements at baseline and month 12 (DMAb) or month 24 (ALN). Abbreviations: ALN, alendronate; BTM, bone turnover marker; CTX-1, serum C-telopeptide; DMAb, denosumab; P1NP, N-terminal propeptide type I procollagen. ^a^At month 18, n = 60.

With denosumab treatment in year 1, the mean percent change in BMD from baseline to month 12 was 5.4%, 3.1%, and 2.7% for the LS, TH, and FN, respectively. After transitioning to alendronate in year 2, the mean percent change in BMD from month 12 to month 24 was 0.5%, 0.5%, and −0.2% at the LS, TH, and FN, respectively. Evaluating the entire study period, participants showed an average gain in BMD above baseline of 5.9%, 3.6%, and 2.5% at the LS, TH, and FN, respectively (Fig. 1). The median percent change in CTX-1 from baseline to month 12, month 18, and month 24 was −69.1%, −64.7%, and −54.8%, respectively. The median percent change in P1NP from baseline to month 12, month 18, and month 24 was −67.7%, −57.0%, and −53.1%, respectively (Fig. 2).

### Participants grouped into those who lost, maintained, or gained BMD in year 2

**Figure 3.**
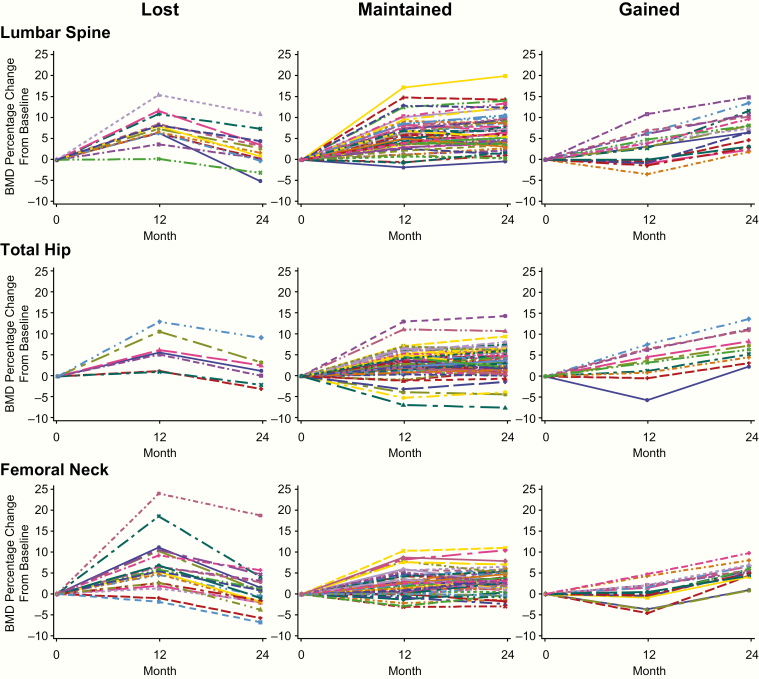
Individual participant BMD changes for groups that lost, maintained, or gained BMD in year 2 on alendronate. Abbreviation: BMD, bone mineral density.

Fig. 3 shows BMD responses for individual participants throughout the study for the groups that lost, maintained, and gained BMD during year 2 on alendronate. Of the 82 participants with BMD measurements available at the LS, 13 (15.9%) lost BMD, 52 (63.4%) maintained BMD, and 17 (20.7%) gained BMD at this site. Of the 92 participants with BMD measurements available at the TH and FN, 7 (7.6%) lost BMD, 75 (81.5%) maintained BMD, and 10 (10.9%) gained BMD at the TH, and 20 (21.7%) lost BMD, 56 (60.9%) maintained BMD, and 16 (17.4%) gained BMD at the FN (Table 3). Of the 82 participants with BMD measurements available at the LS, TH, and FN, only 1 participant (1.2%) lost BMD at all 3 sites.

**Table 3.** Participant Characteristics and BMD Change in Year 2 After Transitioning to Alendronate

	Lumbar Spine (N = 82)			Total Hip (N = 92)			Femoral Neck (N = 92)		
Participants stratified by BMD change category from M12 to M24^a^	Lost	Maintained	Gained	Lost	Maintained	Gained	Lost	Maintained	Gained
n (%)	13 (15.9)	52 (63.4)	17 (20.7)	7 (7.6)	75 (81.5)	10 (10.9)	20 (21.7)	56 (60.9)	16 (17.4)
Baseline characteristics									
Age (years), mean (SD)	63.8 (5.5)	65.5 (7.7)	64.3 (8.2)	66.4 (8.5)	64.5 (7.1)	68.5 (7.5)	67.1 (8.8)	64.2 (6.7)	65.8 (7.2)
BMD T-score, mean (SD)	−1.8 (2.0)	−2.1 (1.1)	−1.9 (0.8)^b^	−2.0 (0.5)	−1.4 (0.7)	−2.2 (0.7)	−1.9 (0.4)	−2.0 (0.5)	−2.2 (0.4)
CTX-1 (ng/mL), mean (SD)	0.49 (0.19)	0.53 (0.29)	0.53 (0.25)	0.63 (0.38)	0.49 (0.25)	0.68 (0.17)	0.52 (0.26)	0.53 (0.27)	0.51 (0.26)
P1NP (µg/mL), mean (SD)	52.8 (9.5)	55.7 (25.1)	58.8 (25.1)	52.4 (20.6)	55.3 (22.8)	63.7 (22.7)	54.4 (16.4)	56.3 (25.2)	56.6 (22.9)
History of fracture (yes), n (%)	7 (53.9)	23 (44.2)	10 (58.8)	4 (57.1)	37 (49.3)	3 (30.0)	7 (35.0)	26 (46.4)	11 (68.8)
M12 and M24 characteristics	**n** = **12**	**n** = **51**	**n** = **14**	**n** = **7**	**n** = **71**	**n** = **10**	**n** = **19**	**n** = **55**	**n** = **14**
% change in BMD from M0 to M12, mean (SD)	7.1 (3.1)	5.9 (3.8)	3.1 (3.9)	6.2 (4.5)	3.0 (3.1)	2.8 (4.0)	7.0 (6.3)	2.7 (2.9)	0.6 (2.6)
	**n** = **12**	**n** = **51**	**n** = **15**	**n** = **7**	**n** = **71**	**n** = **10**	**n** = **19**	**n** = **55**	**n** = **14**
BMD (g/cm^2^) at M12, mean (SD)	1.0 (0.3)	0.9 (0.2)	0.9 (0.1)	0.8 (0.1)	0.8 (0.1)	0.7 (0.1)	0.7 (0.1)	0.7 (0.1)	0.6 (0.1)
	**n** = **12**	**n** = **51**	**n** = **15**	**n** = **7**	**n** = **71**	**n** = **10**	**n** = **19**	**n** = **55**	**n** = **14**
BMD T-score at M12, mean (SD)	−1.2 (2.4)	−1.7 (1.2)	−1.7 (0.8)	−1.7 (0.5)	−1.2 (0.7)	−2.1 (0.6)	−1.5 (0.6	−1.9 (0.6)	−2.2 (0.5)
	**n** = **8**	**n** = **32**	**n** = **11**	**n** = **6**	**n** = **43**	**n** = **5**	**n** = **15**)	**n** = **32**	**n** = **7**
% change in CTX-1 from M0 to M12, mean (SD)	−61.3 (18.9)	−62.4 (24.7)	−52.0 (34.9)	−71.9 (20.2)	−58.6 (26.9)	−67.1 (21.2)	−56.7 (27.3)	−66.4 (22.8)	−44.4 (30.3)
	**n** = **9**	**n** = **33**	**n** = **13**	**n** = **6**	**n** = **47**	**n** = **5**	**n** = **16**	**n** = **33**	**n** = **9**
% change in P1NP from M0 to M12, mean (SD)	−31.2 (32.4)	−47.3 (28.1)	−50.5 (20.9)	−23.2 (36.2)	−48.0 (25.9)	−62.2 (12.2)	−34.1 (33.3)	−55.4 (22.0)	−36.9 (24.2)
	**n** = **13**	**n** = **52**	**n** = **17**	**n** = **7**	**n** = **75**	**n** = **10**	**n** = **20**	**n** = **56**	**n** = **16**
% change in BMD from M0 to M24, mean (SD)	2.1 (4.2)	6.7 (4.2)	7.7 (4.0)^b^	1.7 (4.1)	3.3 (3.2)	7.4 (3.8)	0.8 (5.4)	3.0 (2.8)	5.4 (2.3)
BMD at M24 below M0 value, n (%)	3 (23.1)	1 (1.9)	0 (0.0)^b^	2 (28.6)	5 (6.7)	0 (0.0)	10 (50.0)	8 (14.3)	0 (0.0)
ALN adherence at M24, n (%)	11 (84.6)	38 (73.1)	14 (82.4)	5 (71.4)	56 (74.7)	10 (100)	15 (75.0)	45 (80.4)	11 (68.8)

N = number of participants with BMD values at M12 and M24; n = number of participants in each BMD group with available data.

ALN, alendronate; BMD, bone mineral density; CTX-1, serum C-telopeptide; P1NP, N-terminal propeptide type I procollagen; M, month.

^a^Based on a 3% BMD threshold. A BMD change ≤ −3% indicated lost BMD; > −3% and < 3% indicated maintained BMD; and ≥ 3% indicated gained BMD; ^b^n = 16.

### Participant characteristics and BMD change in year 2

The BMD change in year 2 was similar regardless of baseline characteristics such as age, BMD T-score, baseline levels of BTMs, and history of fracture (Table 3). For all skeletal sites, subjects who lost BMD with alendronate in year 2 had shown a greater percent change in BMD with denosumab in year 1 (Fig. 3, Table 3). At the LS, participants who lost BMD in year 2 had gained an average of 7.1% BMD in year 1, while those who gained BMD in year 2 had gained an average of 3.1% BMD in year 1, and a similar difference was observed at the TH (6.2% vs 2.8%). At the FN, participants who lost BMD in year 2 had gained an average of 7.0% BMD in year 1, while those who gained BMD in year 2 had gained an average of 0.6% BMD in year 1. BMD did not fall below pretreatment baseline in the majority of participants and was observed most often in participants who lost BMD with alendronate. Among participants who lost BMD in year 2 at a given skeletal site, 23.1% fell below their baseline BMD value at the LS, 28.6% fell below their baseline value at the TH, and 50.0% fell below their baseline value at the FN.

While adherence to oral alendronate in year 2 was lower than adherence to denosumab in year 1 (16), there was no numeric trend between alendronate adherence and the BMD response in year 2 (Table 3). Compliance, defined as the percentage of provided alendronate tablets taken, was also investigated in terms of the BMD change in year 2 using quartiles of compliance, and there were no differences in the percent change in BMD in year 2 among the 4 compliance subgroups (data not shown).

### Adverse event summary

Safety analysis was performed for 125 participants who received at least 1 dose of denosumab in year 1 and 110 participants who received at least 1 dose of alendronate in year 2 (Table 4). The adverse event profiles were similar between treatment periods, with 74.4% and 61.8% of participants experiencing adverse events during denosumab and alendronate treatment, respectively. The most frequent adverse events (denosumab year 1, alendronate year 2) included arthralgia (8.8%, 6.4%), pain in extremity (7.2%, 3.6%), back pain (4.0%, 2.7%), and cough (4.0%, 4.5%). Adverse events of fracture were experienced by 1 participant during year 1 and 1 participant during year 2; both fracture events were classified as osteoporotic and nonvertebral. No deaths, osteonecrosis of the jaw, or atypical femoral fractures were reported.

**Table 4.** Summary of Adverse Events

	Denosumab/Alendronate Sequence	
	Denosumab Year 1 (N = 125)	Alendronate Year 2 (N = 110)
Adverse events, n (%)		
All	93 (74.4)	68 (61.8)
Serious	4 (3.2)	4 (3.6)
Adverse events of interest in either treatment group, n (%)		
Arthralgia	11 (8.8)	7 (6.4)
Pain in extremity	9 (7.2)	4 (3.6)
Cough	5 (4.0)	5 (4.5)
Back pain	5 (4.0)	3 (2.7)
Osteoarthritis	2 (1.6)	3 (2.7)
Headache	4 (3.2)	3 (2.7)
Adverse events of fracture,^a^ n (%)	1 (0.8)	1 (0.9)

N = number of participants who received at least 1 dose of investigational product during the corresponding treatment period (safety analysis population); n = number of participants reporting at least 1 adverse event during the corresponding period.

Includes only treatment-emergent adverse events that started on or before the end of the corresponding treatment period.

^a^Reported fractures were classified as osteoporotic and nonvertebral.

## Discussion

Transitioning to alendronate was generally effective at preserving the gain in BMD and suppression of BTMs achieved with 1 year of denosumab treatment. Among the minority of participants who lost BMD at the LS, TH, or FN after transitioning to alendronate, few fell below their pretreatment baseline BMD value, and only 1 participant lost BMD at all skeletal sites. Larger BMD increases in year 1 on denosumab were observed for participants who lost BMD in year 2 on alendronate, while other participant characteristics showed no numeric trend with the BMD response in year 2 on alendronate. These findings demonstrate that most women receive benefit from oral bisphosphonate therapy following denosumab cessation.

In the pivotal phase 3, randomized FREEDOM trial and open-label extension, treatment with denosumab for up to 10 years was associated with a continued increase in BMD, sustained reduction in BTMs, and low incidence of fractures, and was generally well tolerated (9). In head-to-head studies, denosumab treatment led to larger increases in BMD and greater reductions in BTMs compared with alendronate (22, 23), which is in agreement with the results presented here. However, because denosumab is a reversible inhibitor of RANKL, denosumab’s effects on bone turnover are reversible with discontinuation, and cessation of denosumab has been associated with rapid loss of vertebral fracture protection, including multiple vertebral fracture (24). Thus, although denosumab treatment can produce large gains in BMD and significant suppression of BTMs, these effects do not protect patients when therapy is discontinued. For this reason, the use of a “drug holiday” in patients receiving denosumab is not recommended (25). The decision to discontinue denosumab treatment should be accompanied by careful monitoring and use of a follow-on antiremodeling agent.

Limited data are available regarding the optimal post-denosumab bisphosphonate treatment regimen (26). In a small case series evaluating women followed for up to 2 years after the FRAME trial, zoledronic acid (n = 11) infusion after denosumab discontinuation showed 73% to 87% preservation of the gains in BMD at the TH or LS after 1 year, with minimal further BMD loss at any skeletal site in year 2; participants given risedronate (n = 5) showed only partial preservation of BMD (41%–64%) (13, 27). These findings are somewhat inconsistent with a previous, smaller case series showing only minimal efficacy to preserve BMD after denosumab treatment cessation (28). This difference in outcome may be related to the inclusion of participants with different ages, previous osteoporosis treatments, and durations of denosumab treatment, and it should be noted that intravenous zoledronic acid was administered 6 or 8 months after the last denosumab injection. In the current analysis, which included a larger number of participants , 1 year of alendronate treatment following 1 year of denosumab led to maintained or increased BMD at the TH or LS in 84% to 92% of participants. These findings demonstrate the potential for bisphosphonate treatment to prevent reversible bone loss in patients who discontinue denosumab treatment and may suggest that oral alendronate helps maintain BMD in the period immediately following denosumab discontinuation when the effects of treatment on bone turnover have dissipated. Ongoing randomized clinical trials evaluating bisphosphonate use after denosumab discontinuation should provide clarity on the optimal treatment regimen (29, 30).

Although the majority of participants maintained or gained BMD after transitioning from denosumab to alendronate in the current analysis, 15.9%, 7.6%, and 21.7% lost BMD at the LS, TH, and FN, respectively, with only 1 participant losing BMD at all sites. These differences between sites in the response to treatment may be due to differences in the proportions of cortical versus trabecular bone as well as load-bearing parameters. Also, degenerative artifact is likely to have a greater impact on BMD at the LS compared with TH. In general, cortical bone may respond less rapidly to antiresorptive therapies compared with trabecular bone, as cortical bone has less surface area per unit volume of mineralized bone matrix upon which bisphosphonates can be adsorbed (31). Specifically, denosumab is likely to have superior effects on cortical bone as compared with alendronate. In a head-to-head study using high-resolution peripheral computed tomography (HR-pQCT), there was greater reduction in cortical porosity at the radius and tibia in patients receiving denosumab compared with alendronate (32). Supportive data using another technology to measure cortical porosity indicate the efficacy of denosumab in reducing cortical porosity at the hip, a load-bearing site (33). Although the FN is included in the TH region of interest, the FN comprises a much smaller region and provides a less precise measurement than the TH, which is the preferred region of interest for following individual patients. Together, these differences can lead to variability in the clinical response to osteoporosis therapies.

The current analysis sought to better understand patient characteristics linked to loss of BMD after transitioning from denosumab to alendronate treatment, and our results identified the change in BMD with denosumab treatment from baseline to month 12. Specifically, the BMD gain in year 1 on denosumab was numerically higher in individuals who lost BMD in year 2 on alendronate than in those who maintained or gained BMD in year 2. This result may be attributable to “regression to the mean,” a characteristic of imprecision of measurement. Bone remodeling differs between individuals and is influenced by a variety of factors (34, 35). Closure of the remodeling space with antiresorptive treatment in high remodelers might produce greater gains in BMD with treatment. In these individuals, subsequent discontinuation of reversible treatment may result in resumption of the same level of remodeling, which may not be fully inhibited with a bisphosphonate. However, alendronate treatment was able to maintain BMD above the pretreatment baseline level in the majority of women who lost BMD in year 2.

No other baseline or year 1 participant characteristics could consistently identify participants who lost BMD after transitioning to alendronate treatment in this analysis. This finding suggests that all patients, regardless of baseline characteristics and fracture history, may benefit from follow-on therapy with bisphosphonates after discontinuation of denosumab. We did not observe a meaningful effect of adherence to oral alendronate treatment on the change in BMD in year 2 after denosumab discontinuation. However, this may be the result of the overall good adherence in our clinical trial patient population, as compared to clinical practice where adherence can be low. Thus, it is likely that the low rate of nonadherence did not allow us to detect an effect of nonadherence on BMD decline in our patient population. Our findings also stress the importance of BMD monitoring while on treatment to identify those at greatest risk for fracture, as those patients with the largest increase in BMD with denosumab might also be the most vulnerable to BMD loss while receiving alendronate. Unfortunately, in clinical practice, most patients who discontinue denosumab treatment do not receive any osteoporosis treatment in the year following discontinuation; moreover, approximately half of those who begin a prescription medication for osteoporosis after denosumab cessation stop the therapy in the subsequent year (11).

Several limitations of this analysis should be considered. First, denosumab was only administered for 1 year before transition to alendronate. With longer-term denosumab treatment, there will be continued gains in BMD and the potential for greater bone loss after treatment is stopped, and it could be more difficult to preserve bone mass when transitioning to bisphosphonates. Determining the optimal timing, dose, and bisphosphonate medication to administer after denosumab cessation warrants further study. Second, this post hoc analysis had a modest sample size, particularly for the analysis of participants divided into BMD response groups (ie, lost, maintained, and gained). However, the current sample size is larger than that reported in other ad-hoc case series investigating denosumab discontinuation and bisphosphonate follow-on therapy. Third, the analysis was not powered to detect statistical relationships between participant characteristics and their BMD response in year 2, and all analyses were descriptive in nature. Fourth, the effectiveness of alendronate to maintain BMD in this study may have been unrealistically high, given the difficulties with compliance and dosing faced in clinical practice (36). Finally, DXA assessment for this study was not centralized; therefore, BMD results may vary by center.

Denosumab treatment increased BMD at all skeletal sites examined, and the gains in BMD achieved with denosumab were maintained in the majority of participants after transitioning to alendronate. Among participants who lost BMD in year 2 with alendronate, the majority remained above their pretreatment baseline value. Those with larger BMD increases in year 1 often showed greater BMD losses in year 2, with other participant characteristics not related to the response in year 2. These data highlight the need for oral bisphosphonate therapy following denosumab cessation and BMD monitoring of patients transitioning from denosumab to bisphosphonates.

## Abbreviations

BMD: bone mineral density
BTM: bone turnover markers
CI: confidence interval
CTX-1: serum C-telopeptide
DXA: dual-energy x-ray absorptiometry
FN: femoral neck
IQR: interquartile range
LS: lumbar spine
P1NP: N-terminal propeptide type I procollagen
RANKL: receptor activator of nuclear factor kappa-B ligand
TH: total hip

## Additional Information

***Disclosure Summary:*** D.K. has grant/research support from Amgen, AstraZeneca, and Eli Lilly and consultant/speakers’ bureau/advisory activities with Amgen, Pfizer, and Eli Lilly. P.C. has grant/research support from CONACYT (Mexico) Fondos Federales (Mexico); consultant/speakers’ bureau/advisory activities with Amgen, Eli Lilly, and Pfizer; board membership with IOF and National University of Mexico UNAM; and patent licensing for 613227. P.R.E. has grant/research support from Amgen and Eli Lilly and consultant/speakers’ bureau/advisory activities with Amgen, Alexion, and Eli Lilly. M.M. has grant/research support from Amgen and consultant/speakers’ bureau/advisory activities with Amgen and Radius Health. Y.R. has grant/research support from the Korean Ministry of Health and Welfare, Korean Ministry of Science and ICT and consultant/speakers’ bureau/advisory activities with Amgen. A.C., S.H., and R.K.S. are company employees and have stock ownership or royalties with Amgen.

***Data Availability:*** Qualified researchers may request data from Amgen clinical studies. Complete details are available at the following: https://wwwext.amgen.com/science/clinical-trials/clinical-data-transparency-practices/.
